# Design of a Portable Analyzer to Determine the Net Exchange of CO_2_ in Rice Field Ecosystems

**DOI:** 10.3390/s24020402

**Published:** 2024-01-09

**Authors:** Mirko Bonilla-Cordova, Lena Cruz-Villacorta, Ida Echegaray-Cabrera, Lia Ramos-Fernández, Lisveth Flores del Pino

**Affiliations:** 1 Department of Environmental Engineering, Universidad Nacional Agraria La Molina, Lima 15024, Peru; 20170330@lamolina.edu.pe (M.B.-C.); 20170031@lamolina.edu.pe (I.E.-C.); 2 Department of Territorial Planning and Doctoral Program of Engineering and Environmental Sciences, Universidad Nacional Agraria La Molina, Lima 15024, Peru; lenacruz@lamolina.edu.pe; 3 Department of Water Resources, Universidad Nacional Agraria La Molina, Lima 15024, Peru; 4 Research Center for Environmental Chemistry, Toxicology and Biotechnology, Universidad Nacional Agraria La Molina, Lima 15024, Peru; lisveth@lamolina.edu.pe

**Keywords:** sensors, infrared detectors, camera trapping, crop monitoring, rice fields

## Abstract

Global warming is influenced by an increase in greenhouse gas (GHG) concentration in the atmosphere. Consequently, Net Ecosystem Exchange (NEE) is the main factor that influences the exchange of carbon (C) between the atmosphere and the soil. As a result, agricultural ecosystems are a potential carbon dioxide (CO_2_) sink, particularly rice paddies (*Oryza sativa*). Therefore, a static chamber with a portable CO_2_ analyzer was designed and implemented for three rice plots to monitor CO_2_ emissions. Furthermore, a weather station was installed to record meteorological variables. The vegetative, reproductive, and maturation phases of the crop lasted 95, 35, and 42 days post-sowing (DPS), respectively. In total, the crop lasted 172 DPS. Diurnal NEE had the highest CO_2_ absorption capacity at 10:00 a.m. for the tillering stage (82 and 89 DPS), floral primordium (102 DPS), panicle initiation (111 DPS), and flowering (126 DPS). On the other hand, the maximum CO_2_ emission at 82, 111, and 126 DPS occurred at 6:00 p.m. At 89 and 102 DPS, it occurred at 4:00 and 6:00 a.m., respectively. NEE in the vegetative stage was −25 $\mu molCO2\;m^{2}\;s^{-1}$, and in the reproductive stage, it was −35 $\mu molCO2\;m^{2}\;s^{-1}$, indicating the highest absorption capacity of the plots. The seasonal dynamics of NEE were mainly controlled by the air temperature inside the chamber (Tc) (R = −0.69), the relative humidity inside the chamber (RHc) (R = −0.66), and net radiation (R_n_) (R = −0.75). These results are similar to previous studies obtained via chromatographic analysis and eddy covariance (EC), which suggests that the portable analyzer could be an alternative for CO_2_ monitoring.

## 1. Introduction

The increase in the concentration of carbon dioxide (CO_2_) in the atmosphere is one of the main factors responsible for global warming. Currently, CO_2_ levels are at 419 ppm; this represents 150% of the values in the 18th century [1]. This increase is mainly due to anthropogenic activities such as intensive agriculture and changes in land use, among others [2]. Rice (*Oryza sativa*) cultivation extends from tropical to temperate regions [3]. It is the second most important staple food in the world, with an annual production of 740 Mt [4]. It covers 114 countries and an area of 153 Mha in total, or 11% of the world’s arable land [5]. In 2021, Peru produced 3.5 Mt of rice in an area of 417,000 ha [6]. Currently, 90% of rice production is obtained through flood irrigation [7], making it a significant source of methane (CH_4_). Furthermore, nitrous oxide (N_2_O) is mainly generated by nitrification and denitrification processes, which are closely related to soil moisture [8]. Both gases represent approximately 30% and 11%, respectively, of global agricultural emissions [9].

Net Ecosystem Exchange (NEE) is one of the main processes that influence CO_2_ concentration in the atmosphere. Agricultural ecosystems, particularly rice paddies, play a crucial role in carbon absorption. Therefore, it is important to understand their function in carbon (C) flux [10]. For example, Chatterjee et al. [11] monitored lowland paddy fields for one year (dry and wet seasons) using eddy covariance (EC) to evaluate variations in NEE and find a suitable model for the better partitioning of NEE with respect to its components, such as gross primary production (GPP) and ecosystem respiration (Reco). Kumar et al. [12] calculated NEE in rice and wheat systems in the northwest Indo-Gangetic plains. This was the first estimation in a rice–wheat spring sequence using EC. Neogi et al. [13] investigated the characterization of CO_2_ fluxes in tropical lowland rice paddy ecosystems using EC to better understand the environmental impact in terms of C budget in submerged soil.

The land–atmosphere exchange of matter and energy is recorded using EC [14], widely used given its solid theoretical basis. However, it is expensive, difficult to manipulate [15], and susceptible to information gaps [16]. On the other hand, static chambers are used to complement the deficiencies of EC. Nevertheless, they require long monitoring periods [17]. Additionally, the cost of chromatographic analysis for collected gases is high. In this regard, infrared sensors represent an opportunity to solve these challenges. They utilize the non-dispersive infrared (NDIR) principle to measure the concentration of CO_2_ instantly [18]. In addition, they are easy to acquire, manipulate, and program. An automatic estimation and sampling method based on sensors that can replace the conventional methods mentioned and simultaneously increase the efficiency in estimating greenhouse gas (GHG) fluxes is necessary [19].

In this research, a CO_2_ analyzer was designed together with a static chamber to monitor diurnal and nocturnal NEE in rice fields. The objective was to establish a novel, efficient, and dependable method of making resource management decisions for sustainable agricultural practices in Peru.

## 2. Materials and Methods

### 2.1. Site Description

**Figure 1:**
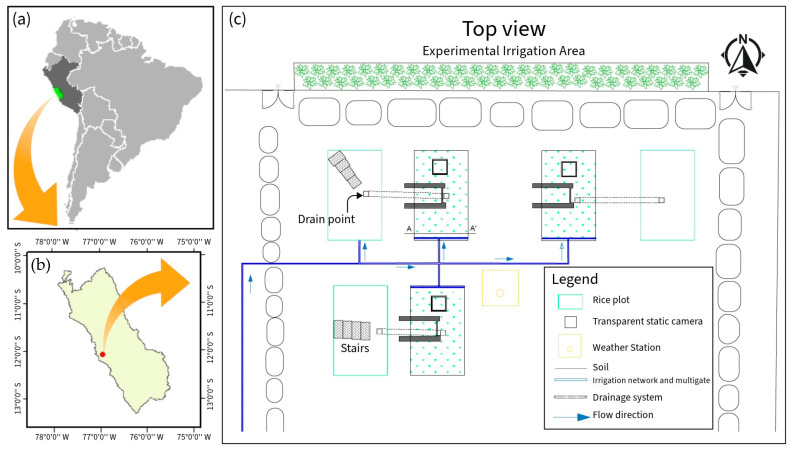
(**a**,**b**) Location of the study area in Lima, Peru. (**c**) AER map.

**Figure 2:**
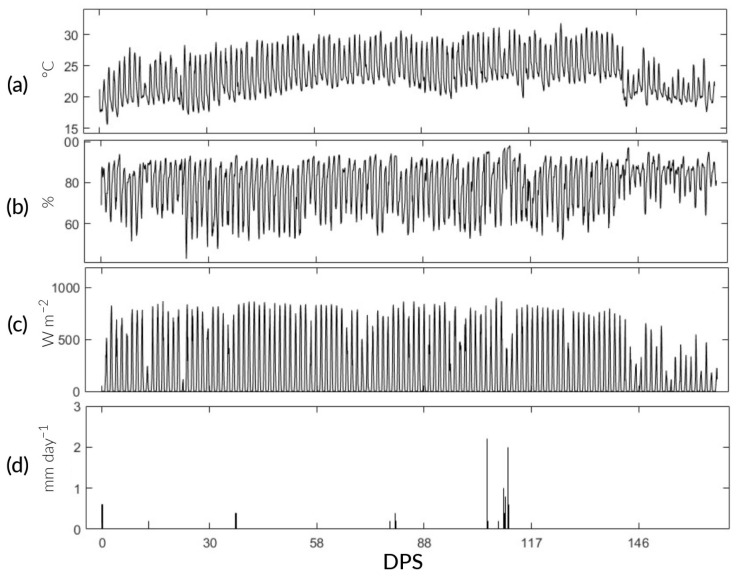
Predominant climatic conditions during the study. (**a**–**d**) Air temperature (T_a_), relative humidity (RH), net radiation (R_n_), and precipitation (pp).

This research was carried out in the “Experimental Irrigation Area” (AER) on the campus of the National Agrarian University La Molina (UNALM), La Molina District, Lima Province, Lima Region (12°04′41″ S, 76°56′45″ W, altitude: 246 m) (Figure 1). During the study, the maximum, minimum, and average temperatures were 32.3, 15.6, and 23.24 °C, respectively. The maximum precipitation was 2.6 mm with an average relative humidity of 77%. The meteorological data were recorded using the automatic station VANTAGE Pro2 Davis, Hayward, CA, USA, located at the AER (Figure 2). In addition, the physicochemical characteristics of the soil are detailed in Table 1.

### 2.2. Design of Portable Analyzer for CO_2_ Monitoring

**Figure 3:**
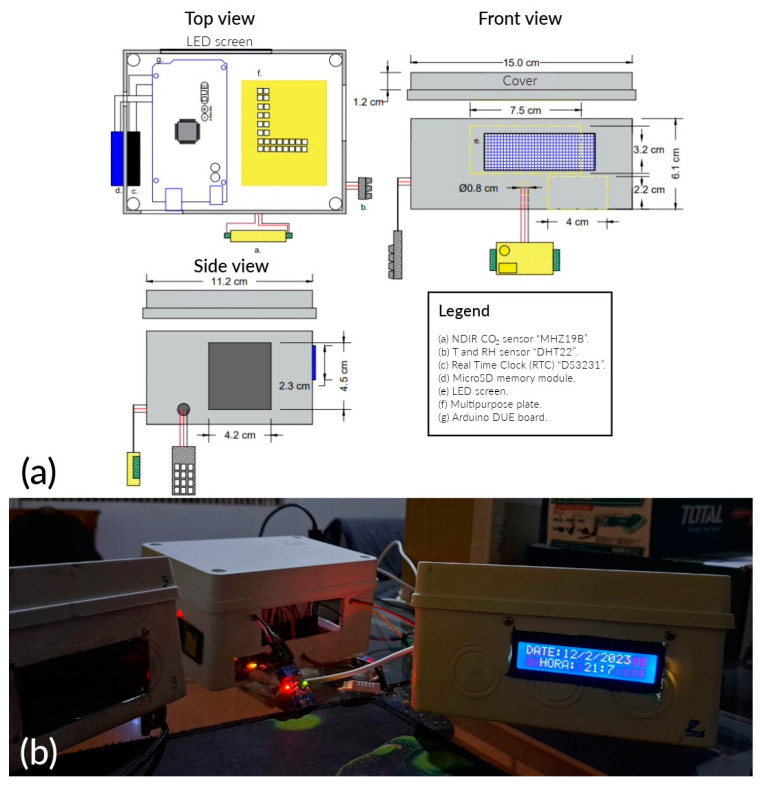
(**a**) Scheme of the portable analyzer for CO_2_ monitoring; (**b**) analyzer finished.

A portable analyzer was designed for CO_2_ monitoring (Figure 3a). Its components are as follows: (a) MHZ19B CO_2_ sensor from Winsen Electronics; its detection range is 0 to 5000 ± 50 ppm. It operates at optimal T_a_ and RH conditions of 0 to 50 °C and 0 to 90%, respectively. (b) DHT22 T_a_ and RH sensor from Aosong Electronics. Its measurement range for T_a_ is −40 to 80 ± 0.5 °C, and for RH, it is 0 to 100 ± 2%. (c) Real-time clock (RTC) module “DS3231” from MMJ Smart Electronics. (d) microSD memory module from Deeoee Electronics. (e) 16 × 2 LED display from Yuxian Electronics. The Arduino DUE board (g) and “Arduino IDE”, both from Arduino CC, were selected as the microcontroller unit and coding system, respectively. The components were soldered onto a multipurpose board (f) to ensure connection with the ARDUINO board. Then, the system was placed in a plastic box measuring 150 × 110 × 80  ${mm}^{3}$. The device was powered by a PHILLIPS 4000 (mAh) portable battery with 5 V of output. The operational analyzer is shown in Figure 3b.

### 2.3. Static Transparent Chamber Design

**Figure 4:**
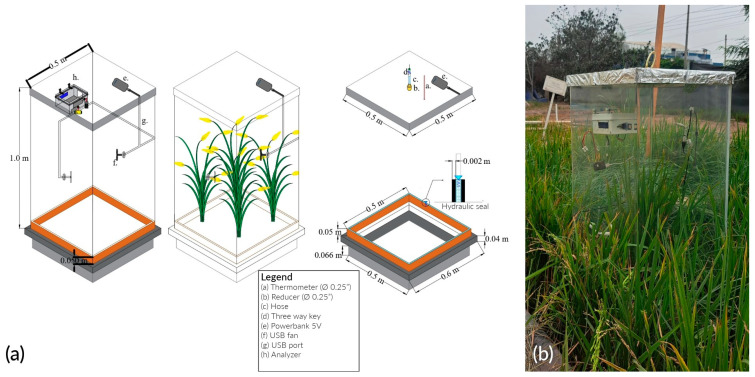
Transparent static chamber: (**a**) scheme; (**b**) disposition.

The monitoring system consisted of a static chamber and a portable CO_2_ analyzer (Figure 4a). The chamber is made of transparent 2 mm thick transparent acrylic, whose dimensions are 1 *×* 0.5 *×* 0.5 $m^{3}$. The gas-mixing system consisted of a portable battery (e) and 2 fans (f), both connected through a Universal Serial Bus (USB) port (g). In addition, the metal base, with dimensions of 0.5 *×* 0.5 *×* 0.15 $m^{3}$, has a 2 mm thick slot. This was installed 6 cm below the soil surface before transplanting permanently. In addition, the analyzer is attached to one of the side faces using a support (h). The finished device is shown in Figure 4b.

### 2.4. Field Management

**Figure 5:**
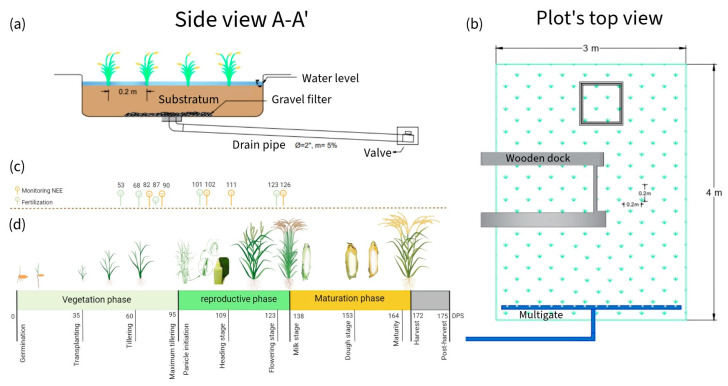
(**a**,**b**) Rice plot’s side and top view, respectively (**c**) Calendar of monitoring and fertilization days (in DPS); (**d**) stages of rice paddy growth.

Three ponds of 3 × 4 × 0.6 $m^{3}$ were installed and lined with geomembrane (Figure 5a,b). The seedbed was prepared on 11 November 2022 and transplanted 35 days post-sowing (DPS). The distribution was five rice seedlings per hill, spaced 20 cm × 20 cm each. The vegetative, reproductive, and maturation phases lasted 95, 35, and 42 DPS, respectively. In total, the crop lasted 172 DPS (Figure 5c). The water regime maintained soil moisture between saturation and a 5 cm depth. Irrigation water came from the Rimac River and was stored in a 25 $m^{3}\;$tank. Its physicochemical characteristics are described in Table 2. The NPK fertilization dose was 230-60-90. In total, 100% of P and K and 50% of N were applied during transplanting. The remaining N was distributed during tillering, floral primordium, and flowering (Figure 5d). The nitrogen sources were urea, diammonium phosphate (DAP), and “Basacote plus 3M”.

### 2.5. Sensor Calibration

**Figure 6:**
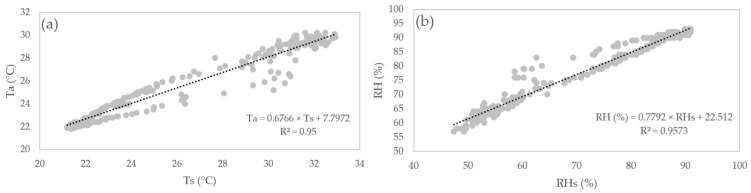
Correlation graph between the DHT22 sensor and the automatic station. (**a**) T_a_; (**b**) RH.

The MHZ19B sensor was calibrated with an automatic reference of 400 ppm by the manufacturer [20]. The DHT22 sensor was calibrated by relating its readings to the hourly data obtained by the automatic weather station for 24 h (Figure 6).

T_a_ is air temperature, T_s_ is the air temperature reading by the sensor, RH is relative humidity, and RHs is the relative humidity reading by the sensor.

### 2.6. Monitoring and Data Collection

Diurnal CO_2_ monitoring in rice plots begins at tillering, the stage of maximum leaf growth. There were 5 days that lasted 24 h each and were carried out simultaneously in the three plots. The preparatory phase began with the attachment of the static camera to the metal base. Then, a water seal was made on the coupling to prevent gas leakage. The analyzer was then placed and turned on in the chamber so that the CO_2_, Ta, and RH readings stabilized for 30 min. Monitoring per se began with closing the chamber and turning on the fans during the first 30 min of each hour. The opposite action was carried out during the remaining 30 min.

### 2.7. Data Processing

Emission fluxes were calculated based on CO_2_ concentration changes $\left(ppm\;{min}^{-1}\right)$. Firstly, linear regression analysis was performed on 30 data [21,22]. Secondly, the CO_2_ emission flux $\left(\mu mol\;m^{-2}\;d^{-1}\right)$ was calculated with Equations (1)–(3).

(1) $$Flux\left(CO_{2}\right)=K\times S$$

(2) $$K=\frac{86400\times P}{10^{6}\times R\times T_{c}}\times \frac{V}{A}$$

(3) $$S=\frac{∆C}{∆t}$$

where K is the accumulation factor of the chamber $\left(mol\;min\;{ppm}^{-1}\;m^{-2}\;d^{-1}\right)$; S is the rate of change in CO_2_ concentration $\left(ppm\;{min}^{-1}\right)$; P is barometric pressure (mbar); R is the ideal gas constant, 0.0831451 $\left(bar\;L\;K^{-1}\;{mol}^{-1}\right)$; T_c_ is the temperature inside the chamber (K); V is the net volume of the chamber $\left(m^{3}\right)$; and A is the net area of the chamber entrance $\left(m^{2}\right)$.

Thirdly, the Michaelis–Menten rectangular hyperbola model was used to calculate NEE [23]. The equation used was (4).

(4) $$NEE=\frac{\left(PPFD\times -P_{max}\right)}{K_{m}+PPFD}-R_{eco}$$

where NEE is the net CO_2_ flux of the rice ecosystem $\left(\mu molCO2\;m^{-2}\;s^{-1}\right)$, PPFD is the photosynthetic photon flux density $\left(\mu molphotons\;m^{-2}\;s^{-1}\right)$, P_max_ is the maximum photosynthetic rate, K_m_ is an adjustment constant, and Reco is the respiration rate of the rice ecosystems $\left(\mu molCO2\;m^{-2}\;s^{-1}\right)$. For this, PPFD, P_max_, and K_m_ data from Yang et al. [21] were used. The daily NEE for each phenological stage is the average of the fluxes from three analyzers. To verify the normality of the data, the Anderson–Darling test was used, which turned out to be non-parametric. Spearman correlation (R) was performed between the environmental variables, NEE, and Reco. Additionally, the Mann–Whitney U test was applied to assess significant differences between the results, at a significance level of 5%.

## 3. Results

### 3.1. Diurnal Variations in NEE

**Figure 7:**
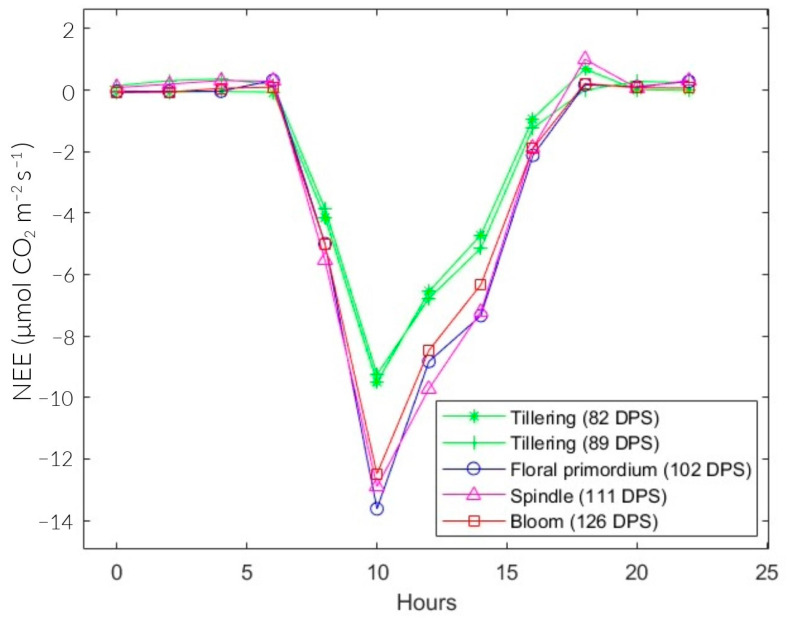
Diurnal NEE for different growth stages of rice fields.

Figure 7 shows the diurnal behavior of NEE in the rice plots. The positive and negative signs indicate the net emission and absorption of CO_2_, respectively. The maximum CO_2_ emission at 89 and 102 DPS was at 4:00 and 6:00 a.m., whose values are 0.361 and 0.318 $\mu molCO2\;m^{2}\;s^{-1}$. At 82, 111, and 126 DPS, it was at 18:00 with values of 0.68, 1, and 0.22 $\mu molCO2\;m^{2}\;s^{-1}$. On the other hand, the maximum CO_2_ assimilation occurred at 10:00 a.m., with values of −9.51, −9.25, −13.63, −12.9, and −12.5 $\mu molCO2\;m^{2}\;s^{-1}$ for 82, 89, 102, 111, and 126 DPS, respectively.

The total NEE was higher during the tillering stage on average (82 and 89 DPS), with −25.07 $\mu molCO2\;m^{2}\;s^{-1}$ on average. In floral primordium (102 DPS), it reached the minimum at −36.14 $\mu molCO2\;m^{2}\;s^{-1}$. Then, it progressively increased during the spindle stage (111 DPS) at −34.98 $\mu molCO2\;m^{2}\;s^{-1}$ and the flowering stage (126 DPS) at −33.83 $\mu molCO2\;m^{2}\;s^{-1}$. Likewise, the seasonal variations in NEE in the vegetative and reproductive phases were −25.07 and −34.98 $\mu molCO2\;m^{2}\;s^{-1}$, respectively (Table 3).

### 3.2. NEE Response to Environmental Factors

**Figure 8:**
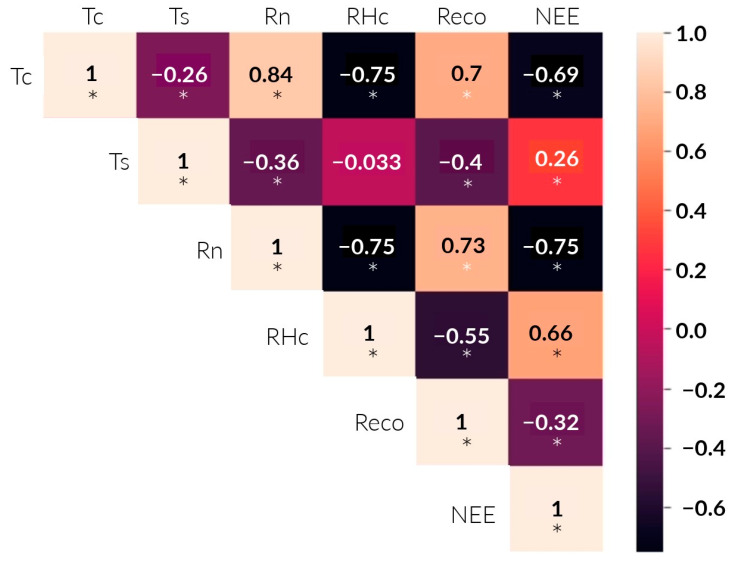
Spearman correlation heat map (R) between NEE, Reco, and environmental variables. Light and dark colors indicate positive and negative correlations, respectively. “*” indicates a significant correlation at the 0.05 level (*p* < 0.05).

The results of the Anderson–Darling test verified the non-normality of the data, except for Ts. Then, the correlations between the environmental factors, Reco, and NEE were analyzed. Coefficients close to 1 and −1 indicate strong positive and negative correlations, respectively (Figure 8). NEE was positively and significantly correlated with RH_c_ (R = 0.66, *p* < 0.05) and T_s_ (R = 0.26, *p* < 0.05). Furthermore, it showed a highly significant negative correlation with Rn (R = −0.75, *p* < 0.05) and T_c_ (R = −0.69, *p* < 0.05). On the other hand, Reco was highly positively associated with T_c_ (R = 0.7, *p* < 0.05) and Rn (R = 0.73, *p* < 0.05). In addition, it showed a significant negative correlation with RHc (R = −0.55, *p* < 0.05) and Ts (R = −0.4, *p* < 0.05).

## 4. Discussion

### 4.1. Diurnal Variation in NEE

**Figure 9:**
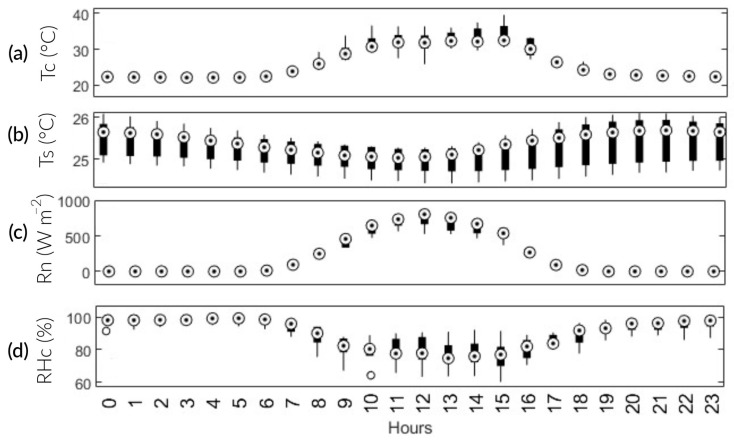
Boxplot graphic: average diurnal variation in the main environmental factors. (**a**) Tc; (**b**) Ts; (**c**) Rn; and (**d**) RHc.

The NEE values during the study are represented in Figure 7. They are positive at night and negative during the day. This behavior is consistent with the results obtained by Bhattacharyya et al. [24], McMillan et al. [25], and Zhang et al. [26]. During daylight hours, the ecosystem functioned as a carbon dioxide (CO_2_) sink, with higher levels of absorption through photosynthesis compared with emissions through respiratory processes. However, during the nighttime, the ecosystem acted as a source of CO_2_, primarily because of Reco [27,28]. In the absence of sunlight, NEE is, on average, 58 times lower than the results of Yang et al. [21] and Bhattacharyya et al. [29]. This decrease can be attributed to the higher levels of RHc during the same period (Figure 9d). As a result, the sensor did not perform at its optimal level. In contrast to portable analyzer technology, the EC methodology used in the aforementioned studies employed open-path NDIR gas analyzers such as the LI-7200, LI-7500, and EC-150. These are specifically designed to measure fluxes in CO_2_, water vapor, and energy below the canopy. Therefore, their prices are excessively higher compare with a portable analyzer. In this study, the “MHZ19B” NDIR sensor was used, which differs in application, precision, and price. However, if optimal operating conditions are guaranteed, the sensor has a high potential for accuracy and practicality.

**Figure 10:**
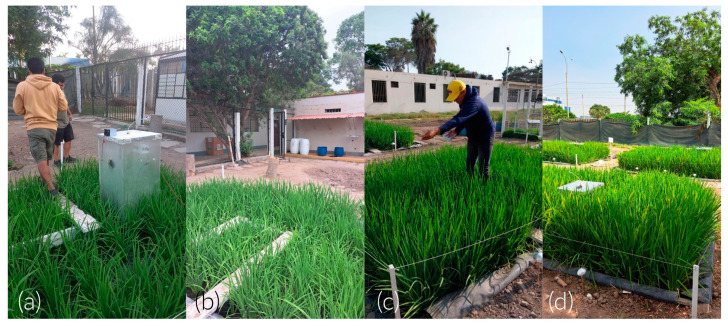
Rice field growth in different stages of the cycle. (**a**–**d**) Tillering (82 and 89 DPS), spindle stage (111 DPS), and flowering (126 DPS).

On the other hand, total NEE during the vegetative phase (−25.2 $\mu molCO2\;m^{2}\;s^{-1}$) was 1.4 times lower than the minimum during the reproductive phase (−35 $\mu molCO2\;m^{2}\;s^{-1}$). Similarly, the maximum diurnal NEE during the reproductive stage (−13 $\mu molCO2\;m^{2}\;s^{-1}$) was 1.4 times higher than during the vegetative stage (−9.4 $\mu molCO2\;m^{2}\;s^{-1}$). This is consistent with Yang et al. [21], who determined that the maximum absorption during the vegetative and maturation phase was approximately 1.5 times lower than in the reproductive phase. In the vegetative phase, CO_2_ assimilation is limited because the plant is in the growth stage (Figure 10a,b). In the reproductive stage, complete development is observed, leading to maximum absorption. In the maturation stage, senescent leaves fall and add organic matter to the soil. Additionally, the plots are drained in preparation for the harvest phase. These two processes gradually increase CO_2_ emissions until the crop is harvested (Figure 10c,d). This behavior is similar to the results of Chen et al. [30].

### 4.2. NEE, Reco, and Their Interactions with Environmental Variables

The results for Reco showed a strong positive correlation with Tc and Rn. It is worth noting that these variables are strongly and positively related (R^2^ = 0.84). According to previous studies, Tc is an important factor in CO_2_ emissions from agricultural ecosystems [30,31]; the same applies to Rn. As Rn and Tc intensify throughout the day, root and microbial activity emits CO_2_ into the atmosphere. The maximum Reco occurs at 12:00 p.m. Nevertheless, photosynthetic activity is higher than Reco. In comparison with Bao et al. [27], it was observed that Reco had a weak negative correlation with Ts, possibly influenced by soil texture, ecosystem type, and water regime. In contrast, the seasonal variation in NEE was negatively related to Tc and Rn. Rn plays a crucial role as the primary energy source for plant metabolism. Consequently, when there is an increase in available energy, plants will absorb more CO_2_ (Figure 9c). These findings line up with the research conducted by Liu et al. [31].

Regarding Tc, the results are consistent with those of Bhattacharyya et al. [29] and Morales [32]. They found an inverse relationship between temperature and CO_2_ assimilation after surpassing 34 °C. This is because the rubisco enzyme, which is essential in CO_2_ fixation, is susceptible to thermal stress. Thus, the temperature inside the chamber exceeded this threshold at 82 DPS between 10:00 a.m. and 3:00 p.m. This may be a factor in why NEE is at its maximum throughout the season. Additionally, between 2:00 p.m. and 6:00 p.m., there is a 3.9-fold increase in CO_2_ emissions (Figure 9a). There is a weak positive correlation between NEE and Ts. Ts, in turn, has a weak negative correlation with Tc. This partially agrees with Liu et al. [31], as they did find a significantly high effect between Tc and Ts.

### 4.3. Comparison with Previous Studies

Information about the environmental and field management conditions from other authors is summarized in Table 4, as they influence CO_2_ absorption (Chen et al. [30] and Li et al. [33]).

**Figure 11:**
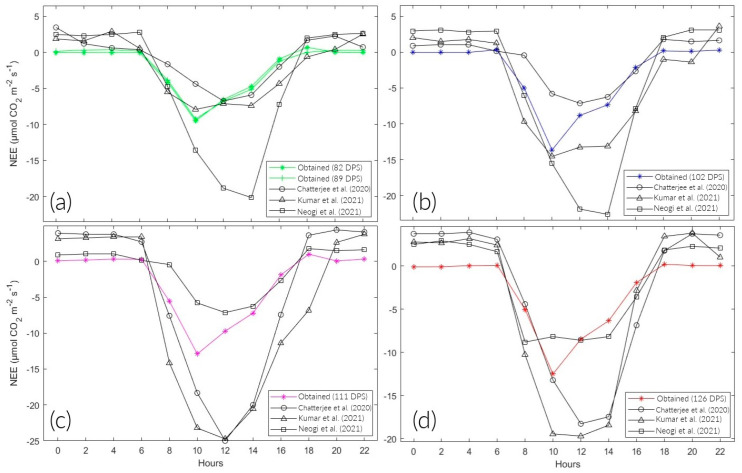
Comparison of the diurnal variation in NEE in different stages of the rice phenological cycle. (**a**–**d**) Tillering, floral primordium, spindle stage, and flowering, respectively, cited in Chatterjee et al. [11], Kumar et al. [12] & Neogi et al. [13].

The results (Figure 11, Table A1, Table A2, Table A3 and Table A4) indicate a lower capacity for CO_2_ absorption than Chatterjee et al. [11], Kumar et al. [12], and Neogi et al. [13]; possibly influenced by climatic conditions. The study was under the conditions of a hot desert (Bwh) climate because of the permanent presence of the South Pacific anticyclone in northern Chile. On the other hand, Chatterjee et al. [11] and Neogi et al. [13] carried out their studies at the ICAR—National Rice Research Institute (NRRI) in India; they recorded climatic conditions typical of the tropical savanna type (Aw). For their part, Kumar et al. [12] at the Indian Agricultural Research Institute (IARI) in Dehli, India, were under hot semiarid climate conditions (Bsh).

Regarding pp during the rice season, a total of 13.4 mm was recorded, despite the influence of the cyclone “Yaku,” which coincided with 111 and 126 DPS (Figure 2d). In comparison with Chatterjee et al. [11] and Neogi et al. [13], whose average annual pp was 1500 mm. 75 and 80% was happened between June and September. Kumar et al. [12], whose studies were carried out in Delhi, recorded 1198 mm during the kharif season for rice cultivation when most of the rains occur from July to September because of the southwest monsoon.

Regarding Tc, it ranged from 21.82 to 33.9 °C on average. On the other hand, Ts fluctuated between 24.95 and 25.51 °C on average. This study was carried out in the months of February to May during the summer season when the cold phase of the El Niño Southern Oscillation (ENSO) is also influential. Chatterjee et al. [11] recorded average annual maximum and minimum temperatures of 39.2 and 22.5 °C. Kumar et al. [12] recorded Ta and Ts values between 31.8 to 38.2 °C and 27.7 to 28.9 °C, respectively. Neogi et al. [13] recorded a progressive increase in temperature as the vegetative cycle of rice continued. From the vegetative stage to harvest, the average temperatures were 23.6 to 33.5 °C, respectively.

However, there were variations in the irrigation techniques used. This study had a maximum water depth of 5 cm during the entire study period. Chatterjee et al. [11] used a higher water regime in three units. Kumar et al. [12] irrigated their crops only when the moisture content fell below the saturation level. In turn, the irrigation regime of Neogi et al. [13] resulted in a sheet of 7–10 cm. According to Yang et al. [22], NEE is sensitive to field management strategies, with water management being one of the most important factors. In addition, soil CO_2_ emissions decrease when flooded with water, as this reduces the diffusivity of the upper layer of soil [34]. These anoxic conditions decrease soil biological activity, as mentioned by Bao et al. [27] and Liu et al. [31]. The result obtained from the Mann–Whitney U test shows that the NEE values did not present a significant difference between the analyzed studies (*p* > 0.05). Therefore, the CO_2_ analyzer generally performed optimally throughout the 24 h of monitoring and throughout the entire study period. However, more research is needed to consider it a reference method (Table 5).

### 4.4. Portable Analyzer Performance

**Figure 12:**
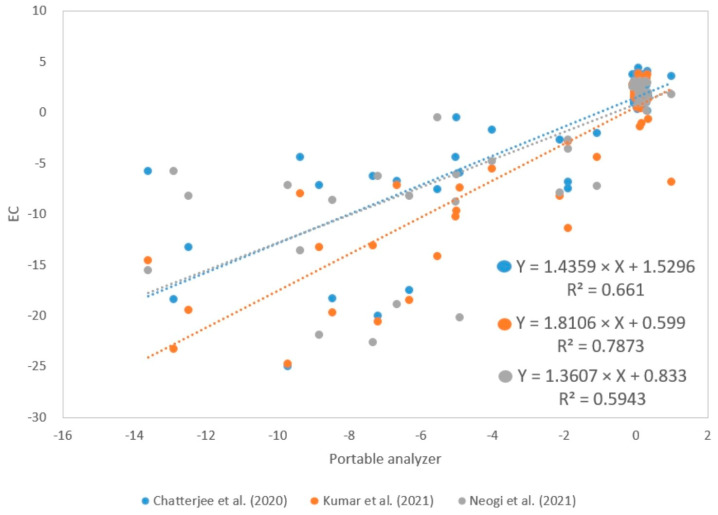
Determination coefficients between NEE values (µmolCO2 m^2^ s^−1^) calculated with the portable analyzer and EC by Chatterjee et al. [11], Kumar et al. [12] & Neogi et al. [13]; respectively.

The MHZ19B sensor has a response time of less than 60 s, so the analyzer was programmed with a response time of one minute to perform a better analysis. On the other hand, open-path analyzers such as LI—7500A and LI—7550 have selectable response times of 0.1, 0.05, and 0.0025 s [35]. In addition, a regression analysis was performed between NEE fluxes calculated from data collected with the portable analyzer and NEE fluxes calculated using EC by Chatterjee et al. [11], Kumar et al. [12], and Neogi et al. [13]. The determination coefficients have values of 0.661, 0.7873, and 0.5943, respectively (Figure 12). These values can be improved if an additional calibration method is taken into consideration and by improving the Tc and RHc conditions. Furthermore, the sensitivity of the sensors is also an important factor to consider since the MHZ19B has a sensitivity of ±50 ppm compared with LI—7500A, with ±0.11 ppm [35].

## 5. Conclusions

A static chamber with a portable CO_2_ analyzer was designed and implemented. It is an economical, simple, and effective alternative to traditional NEE calculation methods. It is a useful tool for making decisions about resource management in agricultural practice in Peru. Rice plots acted as a CO_2_ sink from 6:00 a.m. to 6:00 p.m. and as a CO_2_ source during the remaining period. The minimum NEE values at 82, 89, 102, 111, and 126 DPS were −9.51, −9.25, −13.63, −12.9, and −12.5 $\mu molCO2\;m^{2}\;s^{-1}$; the maximum NEE values for the same dates were 0.68, 0.36, 0.32, 1, and 0.22 $\mu molCO2\;m^{2}\;s^{-1}$, respectively.

The total seasonal NEE values were −25.2 and −34.98 $\mu molCO2\;m^{2}\;s^{-1}$ for the growth and reproductive stages, respectively. This represents a difference of 1.4 times between the mentioned stages. On the other hand, NEE was mainly influenced by Rn (R = −0.75), Tc (R = −0.69), and RHc (R = 0.66). NEE was negative throughout the rice growth period, demonstrating that the rice field acted as a net CO_2_ sink. The results did not show a significant difference compared with previous studies, indicating the optimal performance of the analyzer. Furthermore, differences in CO_2_ absorption can primarily be attributed to the type of crop, irrigation management, and climatic and soil conditions. These results are similar to previous studies obtained via chromatographic analysis and eddy covariance (EC), which suggests that the portable analyzer could be an alternative for CO_2_ monitoring.

## Figures and Tables

**Table 1:** Physicochemical characteristics of the soil in the study area.

Variables	Value
Texture	Loam
$\sigma \;(dS\;m^{-1})$	0.37
pH	7.96
C.I.C$\;(mEq\;{100\;g}^{-1})$	10.40
S.O.M. (%)	3.65
Apparent density$\;(g\;{cm}^{-3})$	1.318
Real density$\;(g\;{cm}^{-3})$	2.74
Porosity (%)	51.89
Field capacity$\;({cm}^{3}\;{cm}^{-3})$	19.91
Wilting point $\left({cm}^{3}\;{cm}^{-3}\right)$	13.91
CaCO_3_ (%)	4.02
P (ppm)	72.4
K^+^ (ppm)	208
Total N (%)	0.21

σ = electric conductivity, pH = hydrogen potential, C.I.C = cation exchange capacity, S.O.M. = soil organic matter, CaCO_3_ = calcium carbonate, P = phosphorus, K^+^ = potassium ion, Total N = total nitrogen.

**Table 2:** Physicochemical characteristics of water.

Variables	Value
pH	8.2
σ $\left(dS\;m^{-1}\right)$	0.67
Ca^2+^ $\left(meq\;L^{-1}\right)$	4.38
Mg^2+^ $\left(meq\;L^{-1}\right)$	0.68
Na^+^ $\left(meq\;L^{-1}\right)$	1.76
K^+^ $\left(meq\;L^{-1}\right)$	0.17
Cl^−1^ $\left(meq\;L^{-1}\right)$	1.57
CO_3_^2−^ $\left(meq\;L^{-1}\right)$	0.10
HCO_3_^2−^ $\left(meq\;L^{-1}\right)$	3.01
SO_4_^2−^ $\left(meq\;L^{-1}\right)$	2.13

pH = hydrogen potential, σ = electric conductivity, Ca^2+^ = calcium ion, Mg^2+^ = magnesium ion, Na^+^ = sodium ion, K^+^ = potassium ion, Cl^−1^ = chloride ion, CO_3_^2−^ = carbonate ion, HCO_3_^2−^ = bicarbonate, ion SO_4_^2−^ = sulfate ion.

**Table 3:** Diurnal NEE (µmolCO2 m^2^ s^−1^) for different growth stages in rice fields.

Hour	PPFD Yang et al. [20]	DPS				
		82	89	102	111	126
0	10	−0.061 ± 0.09	0.143 ± 0.25	−0.034 ± 0.17	0.081 ± 0.19	−0.084 ± 0.15
2	10	−0.069 ± 0.05	0.309 ± 0.29	−0.041 ± 0.05	0.194 ± 0.25	−0.065 ± 0.19
4	10	−0.041 ± 0.12	0.361 ± 0.39	−0.036 ± 0.04	0.313 ± 0.47	0.059 ± 0.4
6	10	−0.064 ± 0.03	0.171 ± 0.21	0.318 ± 0.3	0.290 ± 0.47	0.097 ± 0.5
8	200	−4.148 ± 0.17	−3.860 ± 0.54	−4.997 ± 0.59	−5.550 ± 0.36	−5.021 ± 0.28
10	600	−9.511 ± 0.08	−9.245 ± 0.54	−13.626 ± 0.54	−12.901 ± 1.52	−12.489 ± 1.12
12	400	−6.555 ± 0.53	−6.777 ± 0.91	−8.829 ± 1.29	−9.720 ± 0.32	−8.468 ± 0.35
14	300	−4.710 ± 0.9	−5.141 ± 0.63	−7.336 ± 0.48	−7.202 ± 0.36	−6.330 ± 0.28
16	100	−0.928 ± 0.24	−1.233 ± 0.77	−2.123 ± 0.73	−1.881 ± 0.69	−1.899 ± 0.8
18	10	0.686 ± 0.89	-	0.171 ± 0.21	1.003 ± 0.6	0.218 ± 0.42
20	10	0.015 ± 0.18	0.280 ± 0.28	0.110 ± 0.09	0.069 ± 0.21	0.074 ± 0.28
22	10	−0.014 ± 0.04	0.247 ± 0.21	0.282 ± 0.24	0.321 ± 0.52	0.069 ± 0.3

**Table 4:** Main environmental characteristics of the studies involved.

Site	Köppen–Geiger Climate Classification	Field Management	Soil Texture	Reference
Cuttack, India	Tropical savanna (Aw)	Flood irrigation. Water depth: 8 cm.	Sandy clay loam	Chatterjee et al. [11]
Delhi, India	Warm semiarid (Bsh)	Conventional puddling.	Loam	Kumar et al. [12]
Cuttack, India	Tropical savanna (Aw)	Flood irrigation. Water depth: 7–10 cm.	Sandy clay loam	Neogi et al. [13]
Lima, Peru	Hot desert (Bwh)	Conventional puddling. Water depth: 5 cm.	Loam	-

**Table 5:** Mann–Whitney U test for the values obtained compared with Chatterjee et al. [11], Kumar et al. [12], and Neogi et al. [13]. “**” indicates that there is no significant difference at the 0.05 level (*p* ≥ 0.05).

References	Tillering		Floral Primordium	Spindle State	Bloom
	82 DPS	89 DDS	102 DPS	111 DPS	126 DPS
Chatterjee et al. [11]	96 **	98 **	99 **	88 **	90 **
Kumar et al. [12]	83 **	85 **	75 **	76 **	89 **
Neogi et al. [13]	87 **	88 **	89 **	96 **	94 **

## Data Availability

The data presented in this study are available on request from the corresponding author.

## Appendix A

**Table A1:** Comparison of the diurnal variation in NEE (µmolCO2 m^2^ s^−1^) in tillering.

Hour	PPFD Yang et al. [21]	DPS		References		
		82	89	Chatterjee et al. [11]	Kumar et al. [12]	Neogi et al. [13]
0	10	−0.061 ± 0.09	0.143 ± 0.25	3.439	1.84	2.459
2	10	−0.069 ± 0.05	0.309 ± 0.29	1.186	1.58	2.295
4	10	−0.041 ± 0.12	0.361 ± 0.39	0.593	2.91	2.459
6	10	−0.064 ± 0.03	0.171 ± 0.21	0.356	0.53	2.787
8	200	−4.148 ± 0.17	−3.860 ± 0.54	−1.660	−5.55	−4.754
10	600	−9.511 ± 0.08	−9.245 ± 0.54	−4.387	−7.93	−13.607
12	400	−6.555 ± 0.53	−6.777 ± 0.91	−6.759	−7.14	−18.852
14	300	−4.710 ± 0.9	−5.141 ± 0.63	−5.929	−7.40	−20.164
16	100	−0.928 ± 0.24	−1.233 ± 0.77	−2.016	−4.36	−7.213
18	10	0.686 ± 0.89	-	1.660	−0.66	1.967
20	10	0.015 ± 0.18	0.280 ± 0.28	2.253	0.40	2.459
22	10	−0.014 ± 0.04	0.247 ± 0.21	0.711	2.51	2.623

**Table A2:** Comparison of the diurnal variation in NEE (µmolCO2 m^2^ s^−1^) in floral primordium.

Hour	PPFD Yang et al. [21]	DPS	References		
		102	Chatterjee et al. [11]	Kumar et al. [12]	Neogi et al. [13]
0	10	−0.034 ± 0.17	0.891	2.03	2.956
2	10	−0.041 ± 0.05	1.040	1.50	3.103
4	10	−0.036 ± 0.04	1.040	1.76	2.808
6	10	0.318 ± 0.3	0.149	1.24	2.956
8	200	−4.997 ± 0.59	−0.446	−9.68	−6.059
10	600	−13.626 ± 0.54	−5.792	−14.55	−15.517
12	400	−8.829 ± 1.29	−7.129	−13.23	−21.872
14	300	−7.336 ± 0.48	−6.238	−13.10	−22.611
16	100	−2.123 ± 0.73	−2.673	−8.23	−7.833
18	10	0.171 ± 0.21	1.782	−1.00	2.069
20	10	0.110 ± 0.09	1.485	−1.39	3.103
22	10	0.282 ± 0.24	1.634	3.60	3.103

**Table A3:** Comparison of the diurnal variation in NEE (µmolCO2 m^2^ s^−1^) in spindle state.

Hour	PPFD Yang et al. [21]	DPS	References		
		111	Chatterjee et al. [11]	Kumar et al. [12]	Neogi et al. [13]
0	10	0.081 ± 0.19	3.939	3.15	0.891
2	10	0.194 ± 0.25	3.788	3.28	1.040
4	10	0.313 ± 0.47	3.788	3.41	1.040
6	10	0.290 ± 0.47	2.727	3.41	0.149
8	200	−5.550 ± 0.36	−7.576	−14.14	−0.446
10	600	−12.901 ± 1.52	−18.333	−23.23	−5.792
12	400	−9.720 ± 0.32	−25.000	−24.75	−7.129
14	300	−7.202 ± 0.36	−20.000	−20.58	−6.238
16	100	−1.881 ± 0.69	−7.424	−11.36	−2.673
18	10	1.003 ± 0.6	3.636	−6.82	1.782
20	10	0.069 ± 0.21	4.394	2.65	1.485
22	10	0.321 ± 0.52	4.091	3.79	1.634

**Table A4:** Comparison of the diurnal variation in NEE (µmolCO2 m^2^ s^−1^) in bloom.

Hour	PPFD Yang et al. [21]	DPS	References		
		126	Chatterjee et al. [11]	Kumar et al. [12]	Neogi et al. [13]
0	10	−0.084 ± 0.15	3.750	2.82	2.513
2	10	−0.065 ± 0.19	3.750	2.69	2.932
4	10	0.059 ± 0.4	3.913	3.19	2.513
6	10	0.097 ± 0.5	3.098	2.44	1.675
8	200	−5.021 ± 0.28	−4.402	−10.26	−8.796
10	600	−12.489 ± 1.12	−13.207	−19.43	−8.168
12	400	−8.468 ± 0.35	−18.261	−19.69	−8.586
14	300	−6.330 ± 0.28	−17.446	−18.43	−8.168
16	100	−1.899 ± 0.8	−6.848	−2.84	−3.560
18	10	0.218 ± 0.42	1.793	3.44	1.885
20	10	0.074 ± 0.28	3.750	3.82	2.304
22	10	0.069 ± 0.3	3.587	1.06	2.094
